# Novel Neohesperidin Dihydrochalcone Analogue Inhibits Adipogenic Differentiation of Human Adipose-Derived Stem Cells through the Nrf2 Pathway

**DOI:** 10.3390/ijms19082215

**Published:** 2018-07-29

**Authors:** Ga Eun Han, Hee-Taik Kang, Sungkyun Chung, Changjin Lim, John A. Linton, Jin-Hee Lee, Wooki Kim, Seok-Ho Kim, Jong Hun Lee

**Affiliations:** 1 Department of Food science and Biotechnology, College of Life Science, CHA University, Seongnam-si 13488, Korea; hke112@naver.com (G.E.H.); jhlee81@cha.ac.kr (J.-H.L.); 2 Department of Family Medicine, College of Medicine, Chungbuk National University, Chungbuk 28644, Korea; kanght0818@gmail.com; 3 Department of Pharmacy, College of Pharmacy and Institute of Pharmaceutical Sciences, CHA University, Seongnam-si 13488, Korea; wjdtjdrbs123@naver.com (S.C.); koryoi0709@gmail.com (C.L.); 4 Department of Family Medicine, Severance Hospital, Yonsei University, Seoul 03722, Korea; yohan@yuhs.ac; 5 Department of Food Science and Biotechnology, Graduate School of Biotechnology, Kyung Hee University, Yongin 17104, Korea; kimw@khu.ac.kr

**Keywords:** adipose stem cells, neohesperidin dihydrochalcone, adipogenic differentiation, reactive oxygen species, Nrf2

## Abstract

Obesity, characterized by excess lipid accumulation, has emerged as a leading public health problem. Excessive, adipocyte-induced lipid accumulation raises the risk of metabolic disorders. Adipose-derived stem cells (ASCs) are mesenchymal stem cells (MSCs) that can be obtained from abundant adipose tissue. High fat mass could be caused by an increase in the size (hypertrophy) and number (hyperplasia) of adipocytes. Reactive oxygen species (ROS) are involved in the adipogenic differentiation of human adipose-derived stem cells (hASCs). Lowering the level of ROS is important to blocking or retarding the adipogenic differentiation of hASCs. Nuclear factor erythroid 2-related factor-2 (Nrf2) is a transcription factor that mediates various antioxidant enzymes and regulates cellular ROS levels. Neohesperidin dihydrochalcone (NHDC), widely used as artificial sweetener, has been shown to have significant free radical scavenging activity. In the present study, (*E*)-3-(4-chlorophenyl)-1-(2,4,6-trimethoxyphenyl)prop-2-en-1-one (CTP), a novel NHDC analogue, was synthesized and examined to determine whether it could inhibit adipogenic differentiation. The inhibition of adipogenic differentiation in hASCs was tested using NHDC and CTP. In the CTP group, reduced Oil Red O staining was observed compared with the differentiation group. CTP treatment also downregulated the expression of PPAR-γ and C/EBP-α, adipogenic differentiation markers in hASCs, compared to the adipogenic differentiation group. The expression of FAS and SREBP-1 decreased in the CTP group, along with the fluorescent intensity (amount) of ROS. Expression of the Nrf2 protein was slightly decreased in the differentiation group. Meanwhile, in both the NHDC and CTP groups, Nrf2 expression was restored to the level of the control group. Moreover, the expression of HO-1 and NQO-1 increased significantly in the CTP group. Taken together, these results suggest that CTP treatment suppresses the adipogenic differentiation of hASCs by decreasing intracellular ROS, possibly through activation of the Nrf2 cytoprotective pathway. Thus, the use of bioactive substances such as CTP, which activates Nrf2 to reduce the cellular level of ROS and inhibit the adipogenic differentiation of hASCs, could be a new strategy for overcoming obesity.

## 1. Introduction

Obesity is a global epidemic known to be associated with diabetes, metabolic syndrome, renal impairments, cancers, and cardiovascular diseases, leading to increased medical costs and serious a public health burden [1,2,3]. Several studies have predicted that future obesity prevalence will gradually increase, though its prevalence has leveled off or been stabilized in several developed countries [4,5]. The pathogenesis of obesity is complicated and associated with multiple factors, including distorted adipogenesis through oxidative stress and inflammation as well as excessive calorie intake and physical inactivity [6].

Human bone marrow-derived mesenchymal stem cells (hMSCs), multipotent adult stem cells, can differentiate into three typical lineages: adipocytes, osteoblasts and chondrocytes [7]. Adipose-derived stem cells (ASCs) are mesenchymal stem cells (MSCs) that are obtained from abundant adipose tissue [8,9]. High fat mass is caused by an increase in the size (hypertrophy) and number (hyperplasia) of adipocytes. Excessive calorie intake over expenditure results in energy accumulation and the hypertrophy of adipocytes through increased volume of cytoplasm and cell membrane area. In addition, the recruitment and proliferation of preadipocytes derived from MSCs, especially adipose-derived stem cells, could lead to chronic inflammation, which results in various pathogenic burdens [10,11].

Reactive oxygen species (ROS) are involved in the modulation of cell functions, such as proliferation, differentiation, and survival [12]. ROS derived from nicotinamide adenine dinucleotide phosphate (NADPH) oxidase in mitochondria appear to have a role in adipogenic differentiation [13,14]. It was demonstrated that increased ROS-induced adipogenesis in adipocytes could be eliminated by an NADPH oxidase inhibitor [14]. On the other hand, it was reported that ROS might upregulate CCAAT-enhancer binding protein-α (C/EBP-α) and peroxisome proliferator-activated receptor-γ (PPAR-γ), resulting in adipogenesis in hMSCs [15]. Sterol regulatory element-binding proteins (SREBPs), common lipogenic transcription factors, regulate the expression of lipogenic enzymes, including fatty acid synthase (FAS) [16]. The activation of SREBP was used to evaluate the lipogenesis status of cells and had a synergistic effect with PPAR-γ on lipogenesis [17,18]. Oxidative stress leads to adipogenesis through the upregulation of C/EBP-α, PPAR-γ, SREBP, and FAS. If it is possible to effectively regulate the ROS that induces adipogenic differentiation and lipogenesis, obesity might be prevented. 

Nrf2 is an important cytoprotective transcription factor that induces the expression of phase II enzymes and phase II detoxifying enzymes, resulting in protective action against oxidative stress and reactive carcinogens [19]. Once cells are exposed to oxidative stress, Nrf2 is released from its anchor protein, Kelch-like ECH-related protein-1 (Keap1), in the cytoplasm and migrates to the nucleus [20]. Translocated Nrf2 induces the transcriptional control of the antioxidant response element (ARE) in the promoter region of a target gene, resulting in the expression of phase II detoxifying enzymes such as NAD(P)H: quinine oxidoreductase-1 (NQO1) and antioxidation enzymes such as heme oxygenase 1 (HO-1) [21]. Therefore, dietary phytochemicals that affect Nrf2 activation could be fundamental antioxidants that enhance cellular antioxidant capacity by inducing the gene expression of phase II antioxidant and detoxification enzymes via the Nrf2 pathway [22]. Many studies have been published on the anti-inflammatory and anti-cancer effects of various dietary phytochemicals that activate Nrf2 [23]. In the same context, dietary phytochemicals that activate Nrf2 should be investigated to determine their potential to prevent adipogenesis and lipogenesis. Many natural molecules can modulate adipogenic differentiation in hASCs. A recent study describes the role of melatonin and vitamin D in the modulation of adipogenesis to prevent lipid accumulation in ASCs [24].

Neohesperidin, found in citrus fruits, is a flavanone glycoside with a strong bitter flavor. Citrus flavonoids are the main effective ingredients of citrus species, including oranges, tangerines, and lemons. Previous studies revealed that citrus flavonoids have various pharmacological effects, including anti-inflammation, anti-cancer, and cardiovascular protection activities [25]. As a strong antioxidant, neohesperidin also has significant free radical scavenging and anti-inflammatory activities [26]. Furno et al. reported that *Citrus bergamia* extract decreased adipogenesis and increased lipolysis [27]. *Citrus bergamia* extract is not a pure compound, consisting of naringin, hesperidin, neohesperidin, and neoeriocitrin. After simple hydrogenation, neohesperidin becomes neohesperidin dihydrochalcone (NHDC), which is widely used as a sweetener. NHDC showed strong free-radical scavenging and Nrf2 activation effects [28]. However, no studies have elucidated whether NHDC can suppress adipogenic differentiation and inhibit obesity development.

**Figure 1:**
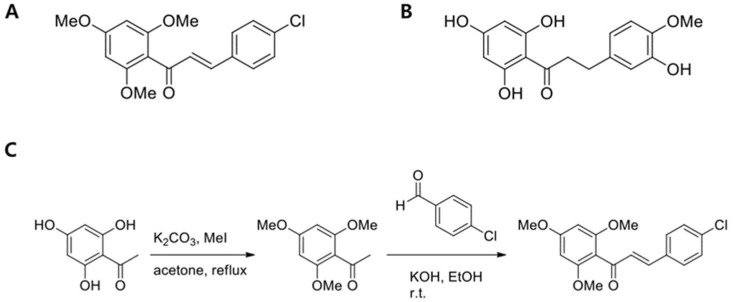
Structures of (*E*)-3-(4-chlorophenyl)-1-(2,4,6-trimethoxyphenyl)prop-2-en-1-one (CTP) (**A**) and neohesperidin dihydrochalcone (NHDC) aglycone (**B**) and a synthetic scheme of CTP (**C**).

Therefore, in this study, we newly synthesized an NHDC analogue, CTP (Figure 1), and we examine the inhibition effects of NHDC and its novel analogue on the in vitro differentiation of hASCs into adipocytes.

## 2. Results

### 2.1. Synthesis of (E)-3-(4-Chlorophenyl)-1-(2,4,6-Trimethoxyphenyl)prop-2-en-1-one (CTP)

CTP was obtained by condensation with commercially available substituted-benzaldehyde. 2,4,6-Trimethoxyacetophenone was synthesized by methylation of 2,4,6-trihydroxyacetophenone with MeI and K_2_CO_3_ in acetone reflux conditions. Spectral data were in agreement with reported data. Chalcone was obtained by aldol condensation of 2,4,6-trimethoxyacetophenone with substituted benzaldehyde (Figure 1).

Yellow solid; m.p. (melting point) 124−126 °C; IR (infra red) ν (neat) cm^−1^: 2835, 1670, 1157, 808, 669 cm^−1^; ^1^H-NMR (CDCl_3_, 300 MHz) δ 7.44 (d, 2H, *J* = 8.6 Hz), 7.31 (d, 2H, *J* = 8.6 Hz), 7.30 (d, 1H, *J* = 15.4 Hz), 6.90 (d, 1H, *J* = 15.4 Hz), 6.13 (s, 2H), 3.84 (s, 3H), 3.75 (s, 6H); HRMS (ESI) Calculated for C_18_H_18_ClO_4_ ([M + H]^+^): 333.0888, found: 333.0889. ν, wavenumber of absorbance; δ, chemical shift.

### 2.2. Cell Viability of hASCs after NHDC and CTP Treatment

**Figure 2:**
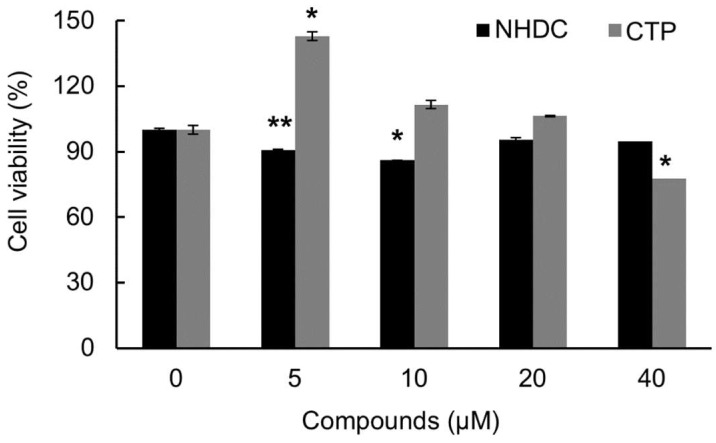
Viability of human adipose-derived stem cells (hASC) in the presence of NHDC and its analogue. Human adipose-derived stem cells (hASCs) were seeded onto a 96-well plate, and the cells were treated with different concentrations (5, 10, 20, and 40 μM) of NHDC and CTP for 5 days. The cell viability was measured by MTS assay. The results are expressed as the mean values ± SD (*n* = 3); * *p* < 0.01, ** *p* < 0.05 as compared to non-treated group (0 μM).

hASCs were seeded at a density of 1 × 10^4^ cells/well on a 96-well culture plate and then grown in 0, 5, 10, 20, or 40 µM of NHDC or CTP for 5 days. Figure 2 shows the cytotoxicity of NHDC and its analogue, CTP. The viability of the hASCs was measured against NHDC and CTP and calculated as a percentage of the untreated control. CTP showed little cytotoxicity up to a concentration of 20 µM. However, it started to show cytotoxicity when the concentration reached 40 µM. Specifically, CTP lowered the viability of the hASCs to 80% at a concentration of 40 µM. Meanwhile, NHDC had no cytotoxicity in hASCs. The treatment concentration was set at 4 µM for the rest of the experiments, much lower than the concentration showing cytotoxicity, because the hASCs needed to be cultured for a long time to reach differentiation. The experimental groups are as follows:

- Control group (Con): Neither NHDC nor CTP treatment during proliferation media treatment only.
- Differentiation group (Diff): Neither NHDC nor CTP treatment during proliferation and differentiation media treatment.
- Experimental group (NHDC of CTP): NHDC or CTP treatment during proliferation and differentiation media treatment.

### 2.3. Inhibition Effects of CTP on the Adipogenic Differentiation of hASCs

**Figure 3:**
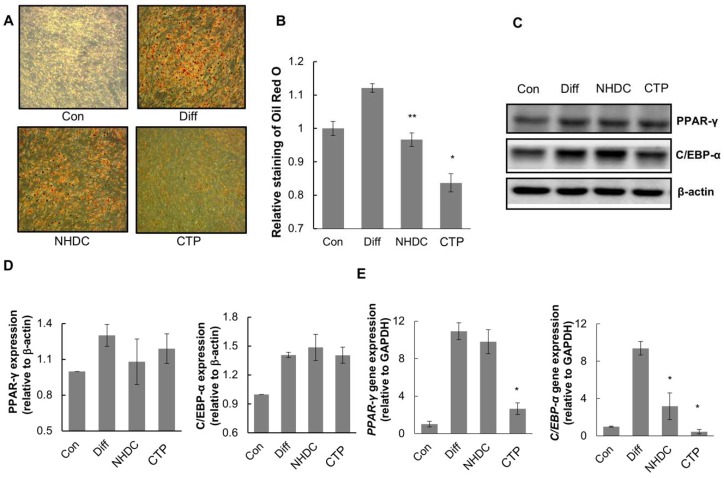
Effect of NHDC and CTP on adipogenic differentiation. Cells were seeded on 12-well plate and cultured for 14 days with or without differentiation medium and compounds. 14 days after culturing in adipogenic differentiation medium or Differentiated hASCs were stained with Oil Red O as described in Materials and Methods (**A**). Lipid accumulation was quantified by Oil Red O uptake and the amount of eluted dye was measured at the absorbance of 510 nm (**B**). Through Western Blotting assay of PPAR-γ and C/EBP-α (**C**) and its quantification, Bands’ densities were analyzed by using ImageJ software (http://rsbweb.nih.gov/ij) (**D**). Quantitative RT-PCR results of *PPAR-γ* and *C/EBP-α* were evaluated (**E**). The results are expressed as the mean values ± SD (*n* = 3); * *p* < 0.01, ** *p* < 0.05 as compared to the differentiation group. Con, control group cultured in growth medium only; Diff, differentiation group cultured in adipogenic differentiation medium.

To examine the inhibition effect of NHDC and its analogue CTP on adipogenic differentiation, we differentiated hASCs into adipocytes using adipogenic differentiation medium and then treated them with NHDC or CTP. The induction of hASC differentiation into adipocytes was evaluated using Oil Red O staining and quantified by the extraction of Oil Red O from the stained cells. In the control group, almost no Oil Red O staining was observed. In contrast, the differentiation group showed the highest Oil Red O staining among all groups (Figure 3A). In the NHDC group, slightly less Oil Red O staining was observed compared with the differentiation group. In the CTP group, the amount of lipid accumulation decreased by 25% compared with the differentiation group (Figure 3B). In addition, we measured the expression of PPAR-γ and C/EBP-α, important adipogenic differentiation markers, as protein levels. The expression of both PPAR-γ and C/EBP-α in the CTP group was decreased slightly compared to the differentiation group (Figure 3C,D). Then, quantitative-PCR was also performed to measure the *PPAR-γ* and *C/EBP-α* mRNA expression to see the gene level effect. The results showed that CTP significantly reduced the expression of *PPAR-γ* and *C/EBP-α* mRNA compared to differentiation group, which is in concordance with the Oil Red O staining analysis (Figure 3E). These results indicate that NHDC analouge could inhibit the adipogenic differentiation of hASCs through down-regulating PPAR-γ and C/EBP-α.

### 2.4. Anti-Lipogenic Effect of CTP during Adipogenic Differentiation of hASCs

**Figure 4:**
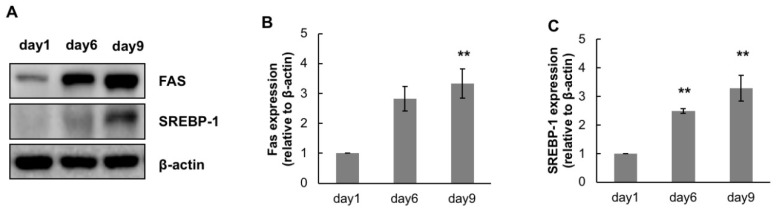
Changes in expression of lipogenic markers over time. The expression of fatty acid synthase (FAS) and SREBP-1, markers for lipogenesis, were measured by Western Blotting at two-time points. To see the lipogenesis in early stage of adipogenic differentiation, hASCs were harvested at 6 days after adipogenic induction. Meanwhile, the lipogenesis in late stage of adipogenic differentiation was investigated at 9 days after adipogenic induction (**A**). Bands were densitometrically analyzed by using ImageJ software (http://rsbweb.nih.gov/ij) (**B**,**C**). The results are expressed as the mean values ± SD (*n* = 3); ** *p* < 0.05 as compared to the control group (day1); day 1, hASCs were cultured in growth medium only; day 6 and day 9, hASCs were cultured in adipogenic differentiation medium for 6 or 9 days, respectively.

Before observing the anti-lipogenic effects of CTP, we investigated changes in the expression of intracellular lipogenic markers over time after treatment with adipogenic differentiation medium. The expression of FAS and SREBP-1, intracellular lipogenic markers, was observed at days 6 and 9 after treatment with adipogenic differentiation medium. To see the early stage of adipogenic differentiation, hASCs were harvested 6 days after adipogenic induction. Late stage adipogenic differentiation was investigated 9 days after adipogenic induction. The expression of intracellular lipogenic markers increased as differentiation proceeded. In particular, the expression of lipogenic markers showed a remarkable increase during the late stage of adipogenic differentiation (Figure 4).

**Figure 5:**
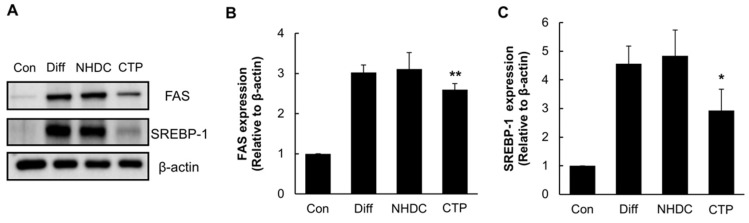
Effect of NHDC analogue on lipogenesis during the adipogenic differentiation. hASCs were treated with NHDC or its analogue along with the adipogenic differentiation medium for 14 days. Western Blotting was performed against lipogenic markers including FAS and SREBP-1 (**A**). Densitometric analysis of Western Blotting of FAS (**B**) and SREBP-1 (**C**). Bands were densitometric analyzed by using ImageJ software. The results are expressed as the mean values ± SD (*n* = 3); * *p* < 0.01, ** *p* < 0.05 as compared to the differentiation group. Con, control group cultured in growth medium only; Diff, differentiation group cultured in adipogenic differentiation medium.

To confirm the effects of CTP on lipogenesis at the late stage of adipogenic differentiation, we examined the protein levels of the lipogenic gene products. As shown in Figure 5A, FAS and SREBP-1 expression was upregulated in the differentiation group, and that high level of expression was decreased by CTP but not by NHDC. In the CTP group, FAS expression decreased by 11.6% (Figure 5B), and SREBP-1 expression decreased by 33.3% (Figure 5C). These data indicate that CTP could retard lipogenesis during hASC differentiation.

### 2.5. Effect of CTP on ROS Generation during Adipogenic Differentiation of hASCs

**Figure 6:**
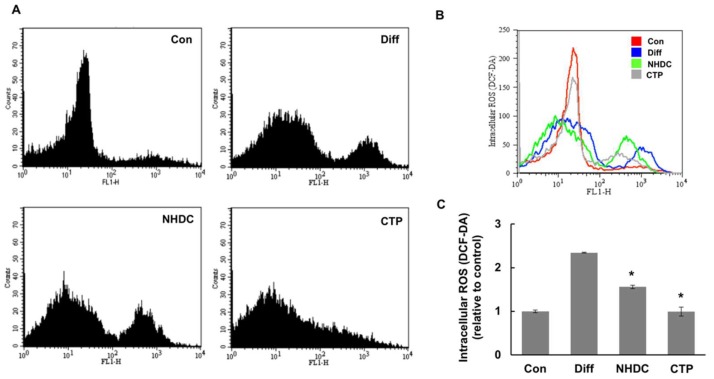
ROS-mediated adipogenic differentiation in hASCs. hASCs were treated with normal growth medium for 5 days and then treated with adipogenic differentiation medium with NHDC or its analogue for 14 days. Meanwhile, the control group was treated with growth medium for 5 days. Intracellular ROS levels were estimated by flow cytometric analysis of DCF fluorescence after staining cells with DCFH-DA. Flow cytometric distribution of DCFDA stained hASCs. (**A**). Flow cytometric distribution among all experimental groups was merged to see the cellular ROS level (**B**). Cell granularity of DCF-fluorescence was evaluated (**C**). The results are expressed as the mean values ± SD (*n* = 3); * *p* < 0.01 as compared to the differentiation group (Diff). Con, control group cultured in growth medium only; Diff, differentiation group cultured in adipogenic differentiation medium.

To measure intracellular ROS levels in differentiated hASCs, we stained the ROS in cells with H_2_DCFDA. Once the staining agent, the acetate ester form of H_2_DCFDA, penetrates a cell membrane, intracellular ROS oxidizes DCFDA to DCF, which generates fluorescence. We used flow cytometry to quantify the level of fluorescence. As shown in Figure 6, the differentiation group showed higher fluorescence levels (more ROS) than the control. Therefore, when hASCs differentiated into adipocytes under the influence of adipogenic differentiation medium, relatively high ROS concentrations accumulated in the cells (Figure 6A,B). Although the fluorescent intensity of the NHDC group was not dramatically weaker than that of the differentiation group, the CTP group showed a strong reduction in fluorescence (Figure 6C). These data suggest that CTP has a strong inhibition effect on ROS generation.

### 2.6. Induction of Nrf2 and Its Downstream Antioxidant Enzymes by CTP in Adipogenic Differentiation of hASCs

**Figure 7:**
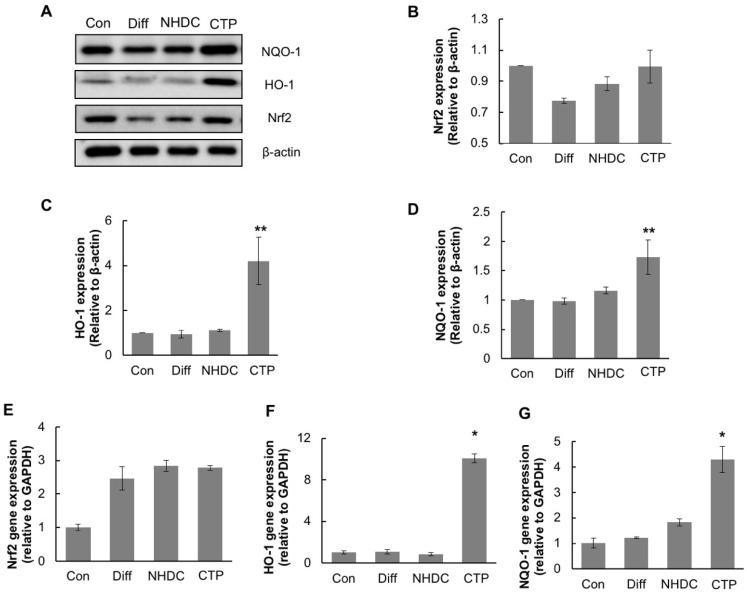
Activation of Nrf2 pathway by NHDC and CTP in hASCs. hASCs, except the control group, were treated with differentiation medium and NHDC and CTP (4μM). The cells were harvested at 14 days after adipogenic induction to examine the expression of Nrf2 and downstream enzymes. Western Blotting was performed against Nrf2 and its downstream antioxidant enzymes (**A**). Quantitative values of the protein expression of Nrf2, HO-1, and NQO-1 were calculated by normalization with β-actin (**B**), (**C**), and (**D**) respectively. mRNA levels of Nrf2 and its downstream enzymes using quantitative RT-PCR were evaluated (**E**), (**F**), and (**G**). The results are expressed as the mean ± SD (*n* = 3); * *p* < 0.01, ** *p* < 0.05 as compared to the differentiation group (Diff). Con, control group cultured in growth medium only; Diff, differentiation group cultured in adipogenic differentiation medium.

To elucidate the cause of the reduction in intracellular ROS production in the CTP group, we investigated the expression of Nrf2, a regulator of antioxidant enzymes (Figure 7). First, we examined the protein levels of the gene products of Nrf2 and its downstream enzymes by Western blot analyses. The expression of Nrf2 protein was slightly decreased in the differentiation group. Meanwhile, in the NHDC and CTP groups, Nrf2 expression was restored to the levels seen in the control group. The expression of HO-1 and NQO-1, downstream antioxidant enzymes of Nrf2, increased significantly in the CTP group (Figure 7C,D). Next, we investigated the effect of CTP on the expression of genes associated with the antioxidant enzymes.The expression levels of mRNA of these genes were measured by quantitative-PCR. The expression of mRNA level of *Nrf2* showed a slight increase in the NHDC and CTP-treated group. In contrast, *HO-1* and *NQO-1* showed significant up-regulation of mRNA expression in the CTP-treated group. Increased levels of both *HO-1* and *NQO-1* mRNA were correlated with the protein levels of gene products (Figure 7F,G). Increases of both protein and mRNA level of *HO-1* and *NQO-1* indicate that CTP could inhibit adipogenic differentiation in hASC through the activation of the Nrf2 pathway, which is due to the regulation of ROS production by upregulating the Nrf2 downstream genes, including *HO-1* and *NQO-1*.

## 3. Discussion

Much attention has been given to the worldwide increase in obesity, which can cause cardiovascular and degenerative diseases. Many researchers in the obesity and adipocyte field have focused on therapeutic methods for weight loss, including dietary, behavioral, and pharmacologic therapy. Unfortunately, to obtain successful results for obesity with dietary and behavioral therapy, sustained efforts are required by individuals, and no evidence suggests that pharmacologic therapy is more effective than other therapies [29]. Therefore, to promote both individual and public health, a new point of view on obesity treatment is necessary. 

The MSCs abundantly present in the abdomen can differentiate into three lineages, chondrogenic, osteogenic, and adipogenic [7]. When adipogenic differentiation occurs, intracellular ROS concentration in MSCs increases [30]. Several reports have demonstrated that Nrf2, a transcription factor regulating antioxidant enzymes, could inhibit adipogenic differentiation in preadipocytes [31,32]. Therefore, the activation of the Nrf2 intracellular antioxidant mechanism might reduce intracellular ROS and inhibit the adipogenic differentiation of hMSCs. Neohesperidin is a strong antioxidant with significant free radical scavenging and anti-inflammatory activity [26]. After simple hydrogenation, neohesperidin becomes NHDC. Based on the antioxidant potential of neohesperidin and NHDC, we synthesized a novel analogue, CTP, and tested it to determine its anti-adipogenic differentiation effects.

In the present study, we investigated whether NHDC and CTP could inhibit hASC differentiation into adipocytes by reducing ROS production through the Nrf2 pathway. CTP reduced lipid accumulation, which was measured with Oil Red O staining, in the differentiation group compared with control. In a cell viability test, CTP showed approximately 20% cell death at 40 μM concentration, but that cytotoxicity did not affect the Oil Red O staining because the concentration applied in that experiment was 4 μM, which has no cytotoxic effect.

We tested whether NHDC and CTP inhibited adipogenic differentiation. The general markers we used to identify adipogenic differentiation in hASCs were lipid accumulation, which we measured by Oil Red O staining, and the expression of PPAR-γ and C/EBP-α [33]. PPAR-γ plays an important role in adipogenic differentiation by regulating the expression of genes involved in adipocyte maturation [34]. It has been reported that adipose-specific PPAR-γ knockout mice fed a high-fat diet had inhibited insulin resistance and decreased body weight compared with controls [35].The increased amount of Oil red O staining and upregulated expression of PPAR-γ observed in the differentiation group indicated the successful adipogenic differentiation of hASCs. In contrast, the Oil Red O staining and PPAR-γ and C/EBP-α expression data indicated no specific adipogenic differentiation in the control group. The CTP group showed reduced lipid accumulation and decreased expression of PPAR-γ and C/EBP-α at both the protein and transcription levels compared to the differentiation group (Figure 3A–E).

SREBP-1, a transcription factor that regulates fatty acid synthesis, is a good indicator of the lipogenic status of a cell. FAS, a target enzyme of SREBP-1, condenses acetyl-CoA and malonyl-CoA to generate long fatty acids [36]. Because overexpressing SREBP-1 enhances lipogenesis, the upregulated SREBP-1 level shown in Figure 4A indicates mature lipogenesis, which correlated with differentiation markers such as Oil Red O staining and PPAR-γ and C/EBP-α expression. In other words, SREBP-1 expression implies lipogenesis in mature adipocytes. Thus, the decreased expression of SREBP-1 seen in the CTP group supports the inhibitive effects of this compound on adipogenic differentiation.

Previous report showed that ROS mediated the adipogenic differentiation in 3T3-L1 cells. H_2_O_2_, exogenous oxidative stress, induced adipogenic differentiation by means of the transcriptional activation of CREB [23]. Because adipogenic differentiation was caused by oxidative stress, we assumed that higher intracellular ROS production would be observed in hASCs when they were differentiated into adipocytes using a common adipogenic differentiation medium. As expected, the flow cytometry results from differentiated hASCs stained with DCFDA showed that the amount of ROS in cells increased as the cells differentiated into adipocytes. Thus, the accumulation of intracellular ROS increases as adipogenic differentiation proceeds in hASCs (Figure 6). Because CTP reduced the production of ROS and the accumulation of lipids, it appears to inhibit adipogenic differentiation by reducing ROS production.

Nrf2, a key transcriptional regulator of various antioxidant enzymes, regulates cellular ROS production [37]. We had already found that CTP reduced intracellular ROS level in hASCs. We thus investigated the expression of Nrf2 to determine whether the reduction in ROS production caused by NHDC and CTP was mediated by the Nrf2 pathway. As shown in Figure 6, the expression of the Nrf2 protein decreased slightly in the differentiation group, whereas in the NHDC and CTP groups, Nrf2 expression was restored to the level of the control group. The expression of HO-1 and NQO-1, two antioxidant enzymes downstream of Nrf2, increased significantly in the CTP group. Nrf2 has been studied extensively, and its cytoprotective mechanism has been described in detail [38]. When ROS accumulates in a cell, Nrf2 in the cytosol translocates into the nucleus, where it interacts with other bZIP transcription factor partners such as small Maf proteins and ATF4 and transactivates the AREs [39,40]. During this process, a time difference could occur between the expression of Nrf2 and the expression of its downstream antioxidant enzymes. It seems that excessive ROS induces the activation of Nrf2, which subsequently induces the expression of downstream antioxidants. While the excessive ROS is being eliminated by the antioxidant enzymes, the cellular Nrf2 level might not increase because it is maintained by ubiquitination [37]. Thus, decreased ROS levels might be regulated by the Nrf2 pathway. As described above, various adipogenic differentiation markers, such as cellular lipid accumulation, PPAR-γ, and C/EBP-α, were downregulated in the CPT group, indicating that CTP could inhibit adipogenic differentiation. In addition, CPT showed strong reduction effects on the ROS levels in hASCs. CPT also activated Nrf2 and its downstream antioxidant enzymes (HO-1 and NQO-1). Notably, those results confirm that reduced ROS levels in hASCs could be regulated by the Nrf2 cytoprotective pathway. Thus, we attribute the inhibition of adipogenic differentiation caused by CTP treatment to the Nrf2-regulated reduction of ROS levels in hASCs.

## 4. Materials and Methods

### 4.1. Chemicals and Reagents

hASCs were obtained from Invitrogen (R7788110) and cultured according to the manufacturer’s instructions in MesenPRO RS^TM^ medium (Invitrogen, Carlsbad, CA, USA) supplemented with growth factor and 2 mM of l-glutamine. Dulbecco’s modified Eagle’s medium (DMEM) and fetal bovine serum (FBS) were obtained from Corning (Acton, MA, USA). Primary antibodies against β-actin (sc-1616), Nrf2 (se-722), HO-1 (sc-10789), NQO-1 (sc-32793), PPAR-γ (sc-7196), and C/EBP-α (sc-9315) were purchased from Santa Cruz Biotechnology (Santa Cruz, CA, USA). For secondary antibodies, donkey anti-goat immunoglobulin G (IgG)-horseradish peroxidase (HRP) (GTX232040-01) was purchased from GeneTex (Insight Biotech, Wembley, UK), goat anti-rabbit IgG-HRP (sc-2301) was purchased from Santa Cruz Biotechnology, and goat anti-mouse–IgG-HRP conjugates (W 402B) were purchased from Promega (Madison, WI, USA).

### 4.2. Synthesis of (E)-3-(4-Chlorophenyl)-1-(2,4,6-Trimethoxyphenyl)prop-2-en-1-one

Unless noted otherwise, all starting materials and reagents were obtained commercially and used without further purification. Tetrahydrofuran was distilled from sodium benzophenone ketyl. Dichloromethane and acetonitrile were freshly distilled from calcium hydride. All solvents used for routine product isolation and chromatography were of reagent grade and glass distilled. Reaction flasks were dried at 100 °C before use, and air- and moisture-sensitive reactions were performed under nitrogen. Flash column chromatography was performed using silica gel 60 (230−400 mesh, Merck, Kenilworth, NJ, USA) with the indicated solvents. Thin-layer chromatography was performed using 0.25 mm silica gel plates (Merck, Kenilworth, NJ, USA). HRMS (High Resolution Mass Spectra) were obtained using a JMS-AX 505WA unit (JEOL, Tokyo, Japan) by an ESI (ElectroSpray Ionization). ^1^H- and ^13^C-NMR (Nuclear Magnetic Resonance) spectra were recorded on a JNM-LA 300 (JEOL, Tokyo, Japan), ADVANCE digital 400 (Brucker, Billerica, MA, USA), ADVANCE digital 500 (Brucker, Billerica, MA, USA) or ECA-600 (JEOL, Tokyo, Japan) in deuteriochloroform (CDCl_3_, Sigma-Aldrich, St. Louis, MO, USA) or deuteriomethanol (CD_3_OD, Sigma-Aldrich, St. Louis, MO, USA). Chemical shifts are expressed in parts per million (ppm, *δ*) downfield from tetramethylsilane and are referenced to the deuterated solvent (CHCl_3_). ^1^H-NMR data are reported in the order; chemical shift, multiplicity (s, singlet; bs, broad singlet; d, doublet; t, triplet; q, quartet; m, multiplet, and/or multiple resonance, numbers of protons, and coupling constants in hertz (Hz). The final compound was purified up to more than 95% purity. The purities were determined by a reverse-phase high-performance liquid chromatography (Waters, Milford, MA, USA., 254 nm) using Eclipse Plus C18 (4.6 × 250 mm) with an isocratic flow (MeOH:H_2_O = 9:1) at 1.5 mL/min.

### 4.3. Cell Culture

hASCs obtained from Invitrogen (R7788110) and cultured according to manufacturers’ instructions. hASCs were cultured in MesenPRO RS^TM^ Medium (Invitrogen, Carlsbad, CA, USA) supplemented with growth factor and 2 mM of l-glutamine and grown at 37 °C in a 5% CO_2_ humidified atmosphere. The culture medium was changed every 3 days and the cells were used in early passages (fourth to seventh). Cells were grown up to 70–80% confluence, rinsed with 1× phosphate buffered saline (PBS) (Dyne BIO, Seongnam, Korea) and detached using 0.25% trypsin-EDTA (Gibco, Paisley, Scotland).

### 4.4. Cell Viability of hASCs with NHDC and CTP

The cytotoxicity of NHDC and CTP on hASCs was estimated using the CellTiter 96 aqueous nonradioactive cell proliferation assay reagent (3-(4,5-dimethylthiazol-2-yl)-5-(3-carboxymethoxyphenyl)-2-(4-sulfophenyl)-2H-tetrazolium, inner salt; MTS) (Promega, Madison, WI). hASCs at a density of 1 × 10^4^ cells/well were cultured in the presence or absence of NHDC and CTP at the concentration of 5, 10, 20, 40 μM/L in the 96-well culture plates. Various concentrations of NHDC and CTP were treated on the first and third days. After 5 days, the cell medium was replaced with 15 μL MTS reagent in 100 μL Basal medium and incubated at 37 °C with 5% CO_2_ for 2 h. The absorbance at 490 nm was measured using a microplate reader (Bio-Tek Instruments, Winooski, VT, USA).

### 4.5. In Vitro Adipogenic Differentiation of hASCs Under NHDC and CTP Treatment

To investigate the effect of NHDC and CTP on adipogenic differentiation, differentiation of hASCs into adipocytes was induced. hASCs were seeded at a cell density of 4.0 × 10^5^ cells/well in the proliferation medium (as previously described, MesenPRO RS^TM^ Medium supplemented with growth factor and 2 mM of l-glutamine) for 5 days, and treated with adipogenic differentiation medium composed of DMEM-high glucose (Gibco, Paisley, Scotland), 10% fetal bovine serum (FBS; Gibco, Paisley, Scotland), 1% penicillin-streptomycin (Pen-strep; Gibco, Grand Island, NY, USA), 10 μg/mL insulin (Gibco, Paisley, Scotland), 500 μM isobutyl-methylxanthine (IBMX; Santa Cruz Biotechnology, CA, USA), 200 μM indomethacin (Tokyo Chemical Industry Co., Ltd., Tokyo, Japan), and 1 μM dexamethasone (Sigma-Aldrich, St. Louis, MO, USA) for another 14 days. 4 μM/L of NHDC and CTP were treated with differentiation medium as needed. Each proliferation and differentiation medium was changed every three days. The cell morphology was observed and photographed under an inverted fluorescence microscope (Olympus CKX53, Tokyo, Japan). hASCs were cultured in proliferation and differentiation medium in the presence or absence of 4 μM/L of NHDC or CTP.

### 4.6. Measurement of Lipid Accumulation during Adipogenic Differentiation of hASCs with and without NHDC and CTP

The accumulation of intracellular lipid droplets was evaluated using Oil Red O (Sigma-Aldrich, St. Louis, MO, USA) staining. The cultured cells were rinsed with PBS and fixed with 4% paraformaldehyde for 15 min at room temperature. After fixation, cells were treated with 60% 2-propanol solution for 10 min. Oil Red O stock solution (0.05% Oil Red O powder in 2-propanol) was diluted with distilled water (volume ratio = 3:2) and filtered through a 0.45 μM syringe filter. Cells were incubated with diluted Oil Red O solution for 30 min at room temperature. After that, the stained cells were washed with PBS three times to remove background. To evaluate the degree of adipogenic differentiation, photomicrographs of the stained cells were captured by optical microscopy. To quantify Oil Red O uptake by the lipids, the dye was eluted with 100% 2-propanol and measured at an absorbance of 510 nm using an ELISA reader (Bio-Tek Instruments, Winooski, VT, USA).

### 4.7. ROS Measurement

The intracellular ROS level was assessed using 2’,7’-dichlorofluorescein diacetate (H_2_DCFDA) (D399; Invitrogen, Carlsbad, CA, USA). H_2_DCFDA is deacetylated intracellularly by esterase, forming H_2_DCF, which is oxidized by ROS to 2’,7’-dichlorofluorescein (DCF), a highly fluorescent compound. Cells were plated at a seeding density of 2.5 × 10^5^ cells/well in a 60 mm culture plate and proceeded adipogenic differentiation with or without 4 μM/L of NHDC and analogue. On 14th days, cells were washed with the PBS and harvested using trypsin-EDTA. After the harvest cells were resuspended and incubated in pre-warmed PBS containing 10 μM DCFDA for 30 min at 37 °C. Then it was transferred to 5 mL FACS tube (SPL Life Science, Pocheon, Korea). Intracellular fluorescence was then quantified using a BD Calibur flow cytometer (Becton Dickinson, NJ, USA) at excitation wavelength of 488 nm and at emission wavelength of 530 nm. Data analyses were based on 10,000 detected events using the Cell Quest software.

### 4.8. Quantitative Reverse Transcription–Polymerase Chain Reaction (qRT-PCR) Analysis

The cell pellet was obtained after being differentiated for 14 days. Total RNA from hASC were purified by Rneasy Mini Kit (Qiagen, Gaithersburg, MD, USA) according to the manufacturer’s protocol. cDNA was synthesized from 1 μg of total RNA by reverse transcription using dNTP mix, Rnase inhibitor (Enzynomics Co., Ltd., Daejeon, Korea), M-MLV Reverse Transcriptase, M-MLV RT 5× Buffer (Promega Corporation, Madison, WI, USA) and random oligo primers. qRT-PCR was performed using SYBR Green 2× Mastermix kit (Messenger of biotechnology, Hanam, Korea) on a BioRad CFX96 Real-Time PCR Detection System instrument (Bio-Rad Laboratories, Hercules, CA, USA) under the following conditions: 10 min at 95 °C, and then 40 cycles of 15 s at 95 °C for denaturation and 60 s at 60 °C for annealing and elongation. Specificity of products was verified by melting curve analysis. The delta-delta-*C*_t_ method was employed to determine the relative gene expression level of gene of interest normalized to the house-keeping gene Glyceraldehyde-3-phosphate dehydrogenase (GAPDH). The sequences of the primers used for differentiation markers and Nrf2 pathway genes are listed in Table 1.

### 4.9. Western Blot Analysis

Cells were washed twice with pre-warmed PBS, lysed with Nonidet P-40 (NP 40) cell lysis buffer (Invitrogen, Carlsbad, CA, USA) and protease inhibitor cocktails (100×) (Barker, Harris, TX, USA) and centrifuged at 14,000 rpm for 10 min (to clarify lysates). Equal concentrations of protein (10 μg) were separated on 12% vertical sodium dodecyl sulfate polyacetylamide gel electrophoresis (SDS-PAGE) and electro-transferred to polyvinylidene fluoride ultrafiltration (PVDF) membranes (Millipore, Billerica, MA, USA), blocked with 3% Bovine serum albumin (BSA) in Tris-buffered saline containing 0.5% Tween-20 (TBS-T) for 1 h, followed by overnight incubation with 1:1000 dilution of the primary antibody. The membranes were washed in several times in TBS-T. And then secondary antibodies were diluted 1:5000 and incubated with membranes at room temperature for 1 h. Immunoreactivity was visualized using an enhanced chemiluminescence detection system with a HRP (ECL STAR Solution, Dyne BIO, Seongnam, Korea) and was analyzed using an LAS-4000 luminescent image analyzer (Fujifilm, Tokyo, Japan).

### 4.10. Statistical Analysis

Data are presented as mean ± standard deviation (SD) or standard error (SE). Data are compared using Student’s *t*-test. All analyses are conducted using SAS statistical software, version 9.1 (SAS Institute Inc, Cary, NC, USA). All statistical tests are two-sided and statistical significance is determined at a *p*-value < 0.01.

## 5. Conclusions

We demonstrated the CTP inhibited adipogenic differentiation by regulation of ROS production through the Nrf2 pathway: NHDC, CTP activated cytoprotective Nrf2 pathway. The activation of Nrf2 by this analogue induced the expression of antioxidant enzymes including HO-1 and NQO-1, resulting in the reduction of ROS production during adipogenic differentiation. Intracellular ROS production appeared to be a key causal effector of adipogenic differentiation. Thus, CTP, having strong effects on Nrf2 activation and ROS reduction in hASC, could be a potential therapeutic tool to overcome obesity.

## Figures and Tables

**Table 1:** Human primers for Polymerase Chain Reaction (PCR).

Gene	Forward	Reverse
*Nrf2*	5’-TCC TCT CCA CAG AAG ACC CC-3’	5’-TCA GGG TGG TTT TGG TTG AA-3’
*HO-1*	5’-ACA TCT ATG TGG CCC TGG AG-3’	5’-TGT TGG GGA AGG TGA AGA AG-3’
*NQO-1*	5’-CAC ACT CCA GCA GAC GCC CG-3’	5’-TGC CCA AGT CAT GGC CCA CAG-3’
*PPAR-γ*	5’-TCT CTC CGT AAT GGA AGA CC-3’	5’-GCA TTA TGA GAC ATC CCC AC-3’
*C/EBP-α*	5’-AGA AAG GGG TGG AAA CAT AGG-3’	5’-GAA AGC TGA GGG CAA AGG-3’
*GAPDH*	5’-AAG GGT CAT CAT CTC TGC CC-3’	5’-ATG ATG TTC TGG AGA GCC CC-3’
